# Applying a hierarchical clustering on principal components approach to identify different patterns of the SARS-CoV-2 epidemic across Italian regions

**DOI:** 10.1038/s41598-021-86703-3

**Published:** 2021-03-29

**Authors:** Andrea Maugeri, Martina Barchitta, Guido Basile, Antonella Agodi

**Affiliations:** 1 Department of Medical and Surgical Sciences and Advanced Technologies “GF Ingrassia”, University of Catania, 95123 Catania, Italy; 2 Department of General Surgery and Medical-Surgical Specialties, University of Catania, 95123 Catania, Italy; 3 Azienda Ospedaliero-Universitaria Policlinico “G. Rodolico - San Marco”, 95123 Catania, Italy

**Keywords:** Infectious diseases, Public health

## Abstract

Italy has experienced the epidemic of Severe Acute Respiratory Syndrome Coronavirus 2, which spread at different times and with different intensities throughout its territory. We aimed to identify clusters with similar epidemic patterns across Italian regions. To do that, we defined a set of regional indicators reflecting different domains and employed a hierarchical clustering on principal component approach to obtain an optimal cluster solution. As of 24 April 2020, Lombardy was the worst hit Italian region and entirely separated from all the others. Sensitivity analysis—by excluding data from Lombardy—partitioned the remaining regions into four clusters. Although cluster 1 (i.e. Veneto) and 2 (i.e. Piedmont and Emilia-Romagna) included the most hit regions beyond Lombardy, this partition reflected differences in the efficacy of restrictions and testing strategies. Cluster 3 was heterogeneous and comprised regions where the epidemic started later and/or where it spread with the lowest intensity. Regions within cluster 4 were those where the epidemic started slightly after Veneto, Emilia-Romagna and Piedmont, favoring timely adoption of control measures. Our findings provide policymakers with a snapshot of the epidemic in Italy, which might help guiding the adoption of countermeasures in accordance with the situation at regional level.

## Introduction

Italy is currently experiencing the epidemic of Severe Acute Respiratory Syndrome Coronavirus 2 (SARS-CoV-2), which emerged in two small geographical areas within the Lombardy and Veneto regions at the end of February, 2020^1^. As of 24 April, Italy had the second highest number of documented SARS-CoV-2 infections in Europe, since there were 189,973 confirmed cases and 25,549 deaths^2^. On 10 March, the Italian government started to react to the epidemic by imposing control measures to the whole country, which included travel restrictions, quarantine and contact precautions^3, 4^. Two weeks later, the government decided to adopt extraordinary measures to further restrict non-essential industrial productions and social interactions^3^. As a consequence, the reported number of new infections started to decline from the last week of March. Although several studies proved the efficacy of these control measures in Italy^4–8^, many of them overlooked the fact that Italy is divided into administrative regions. Indeed, the epidemic situation differed across Italian regions, with several SARS-CoV-2 outbreaks that occurred at different times and with different intensities throughout the Italian territory^2^. For this reason, it was necessary to evaluate the epidemic spread, its consequences, as well as the response to control measures region by region. For instance, a previous study demonstrated how the efficacy of control measures against the SARS-CoV-2 epidemic depended on regional and local factors^9^. Similarly, a network modelling study showed how the heterogeneity between Italian regions was essential to develop effective strategies to control the disease^10^. In this scenario, clustering represents an important data mining methods for uncovering relationships in multivariate datasets^11^. The two most common clustering approaches are hierarchical clustering (i.e. used for identifying groups of similar observations in a dataset) and partitioning clustering (i.e. used for splitting a dataset into several groups)^11^. Previous studies applied clustering analysis to classify SARS-CoV-2 patients according to their socio-demographic, clinical, and behavioural features^12, 13^. We too have previously proposed a simple and immediate approach to categorize Italian regions and provinces based on the prevalence and trend of SARS-CoV-2 cases prior to relaxing national lockdown on 4 May 2020^14^.

Here, we aimed to identify different clusters with similar SARS-CoV-2 epidemic patterns across Italian regions, using a predefined set of indicators. Specifically, we selected indicators reflecting temporal events, intensity and trend of SARS-CoV-2 epidemic in each region, as well as regional characteristics that might affect epidemic spread and patients’ outcomes. Our analysis was limited to the first 2 months of SARS-CoV-2 epidemic in Italy (i.e., from 24 February to 24 April 2020), in order to investigate if such an approach could be helpful to support the development of control measures in the early phase of an epidemic. In the case of multidimensional dataset containing multiple continuous variables, the Principal Component Analysis (PCA) can be used to reduce the dimension of the data into few continuous variables comprising the most important information in the data^15^. Thus, we employed a Hierarchical Clustering on Principal Components approach, which combines three standard methods (i.e. PCA, hierarchical clustering and k-means algorithm) to obtain a better cluster solution^16^.

## Results

We first selected and defined a set of indicators which reflected the beginning of the epidemic, how fast the first peak was reached, intensity and trend during the period considered, and some regional characteristics. The full list of 36 indicators is reported in Table 1. As expected, indicators were strictly interrelated and correlated with each other, with a few exceptions (Figure S1). Indeed, some regional (i.e. mean age, proportion of men, and aging index), temporal (i.e. days to reach maximum number of new infections, hospitalizations, and ICU patients) and trend indicators (i.e. increment of recovered patients on 24 April) did not correlate with others.

**Table 1:** Definition of indicators used to characterize the SARS-CoV-2 epidemic across Italian regions.

Indicator domains	Abbreviations	Definitions
Temporal indicators^a^	d1	Days until first case detected
	d2	Days until first hospitalization occurred
	d3	Days until first patient was admitted to ICU
	d4	Days until first death occurred
	d5	Days until first patient recovered
	d6	Days to reach maximum number of new infections
	d7	Days to reach maximum number of hospitalized patients
	d8	Days to reach maximum number of ICU patients
Intensity indicators	i1	Number of cases on 24 February
	i2	Number of hospitalized patients on 24 February
	i3	Number of ICU patients on 24 February
	i4	Number of cases on 24 April
	i5	Number of new infections on 24 April
	i6	Number of positive patients on 24 April
	i7	Number of hospitalized patients on 24 April
	i8	Number of ICU patients on 24 April
	i9	Number of recovered patients on 24 April
	i10	Number of deaths on 24 April
Trend indicators	t1	Highest number of new infections
	t2	Highest number of hospitalized patients
	t3	Highest number of ICU patients
	t4	Greatest increment of hospitalized patients
	t5	Greatest increment of ICU patients
	t6	Greatest increment of recovered patients
	t7	Greatest increment of deaths
	t8	Increment of new infections on 24 April
	t9	Increment/decrement^b^ of hospitalized patients on 24 April
	t10	Increment/decrement^b^ of ICU patients on 24 April
	t11	Increment of deaths on 24 April
	t12	Increment of recovered patients on 24 April
Regional indicators	r1	Number of tests for SARS-CoV-2
	r2	Number of ICU beds
	r3	Number of residents
	r4	Mean age
	r5	Proportion of male
	r6	Aging index

ICU, Intensive Care Unit; SARS-CoV-2, Severe Acute Respiratory Syndrome Coronavirus 2.

^a^Temporal indicators are computed as the number of days from 24 February, 2020.

^b^These indicators represent daily increment or decrement in relation to different regional scenarios on 24 April 2020.

**Figure 1:**
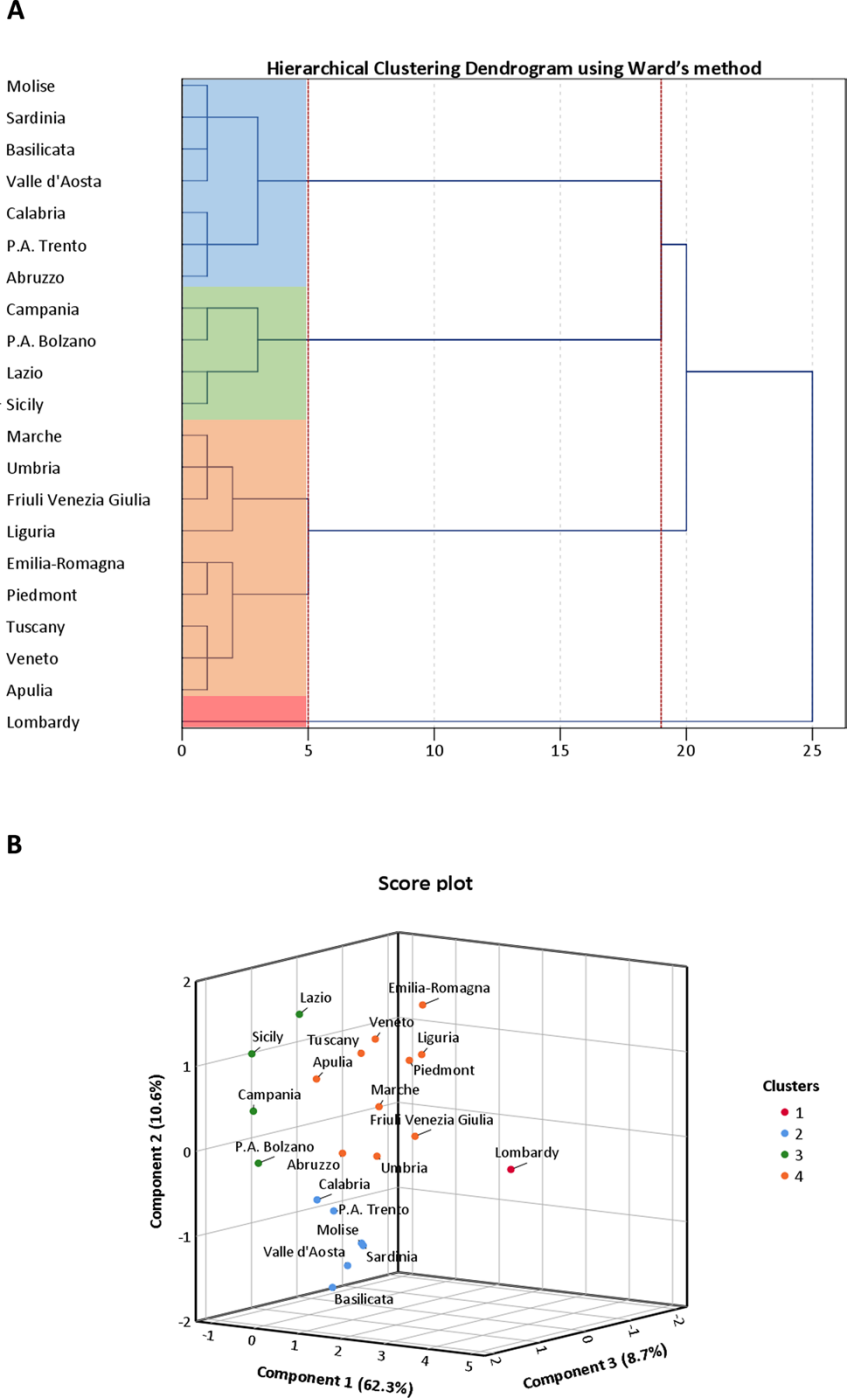
Clustering on Principal Components (PCs). (**A**) Dendrogram of Hierarchical Clustering based on the Ward’s criterion. The height of the branches indicates the dissimilarity between clusters. The dendrogram was partitioned (red dotted lines) to maximize the distance between nodes. Cluster solution is indicated by four colored panels. (**B**) Three-dimensional Score plot illustrating how clusters were distributed on PCs. The number of clusters to be retained was set according to the dendrogram and cluster solution was consolidated by K-means algorithm.

Subsequently, we applied the PCA to reduce this dataset of highly correlated variables into three uncorrelated PCs, which cumulatively explained 81.6% of total variance. The number of PCs to be retained was chosen by visual inspection of the Scree plot (Figure S2) and eigenvalues ≥ 3. In Figure S3a we summarize how each initial variable loaded on PCs: PC1 explained the highest variance among PCs (62.3%) because of high correlations with intensity, trend and regional indicators; PC2 explained 10.6% of variance and was negatively correlated with temporal indicators and positively with regional indicators; PC3 explained 8.7% of variance and was mostly loaded by regional indicators. In Figure S3b we present a three-dimensional Score plot, which illustrates how Italian regions were distributed on PCs. Notably, Figure S3b does not indicate a clear separation in the data but points out that Lombardy was a potential outlier. Based on the hierarchical clustering, we obtained the dendrogram depicted in Fig. 1A, which facilitates the interpretation of the structure of data. As expected, Lombardy separated itself entirely from all the other regions, which instead tended to be grouped into three clusters. It is important to note that more information can be deduced from the dendrogram, such as the dissimilarity between different regions, which is represented by branch size that links them. For instance, even within the same cluster, Sicily appeared to be closer to Lazio than Campania and province of Bolzano. We further consolidated this partition by applying a K-means algorithm with a predefined number of four clusters. In Fig. 1B we present anew the Score plot, in which Regions were assigned to each cluster obtained by K-means clustering. Except for Abruzzo, all the other regions maintained the same allocation of hierarchical clustering. It is worth mentioning that we used several indicators that were not adjusted for the number of residents of each region. On the other hand, however, it is also important to note that we included the number of residents and other regional indicators in the dataset before applying the PCA. This allowed us to obtain a reduced dataset of only three uncorrelated PCs explaining most of total variance. With this in mind and to avoid potential bias due to the different number of residents across Italian regions, we repeated the steps above using a dataset in which intensity and trend indicators were normalized to 100,000 residents. Anyway, the final clustering solution depicted in Figure S4 did not differ from that obtained using non-normalized indicators (Fig. 1B).

**Figure 2:**
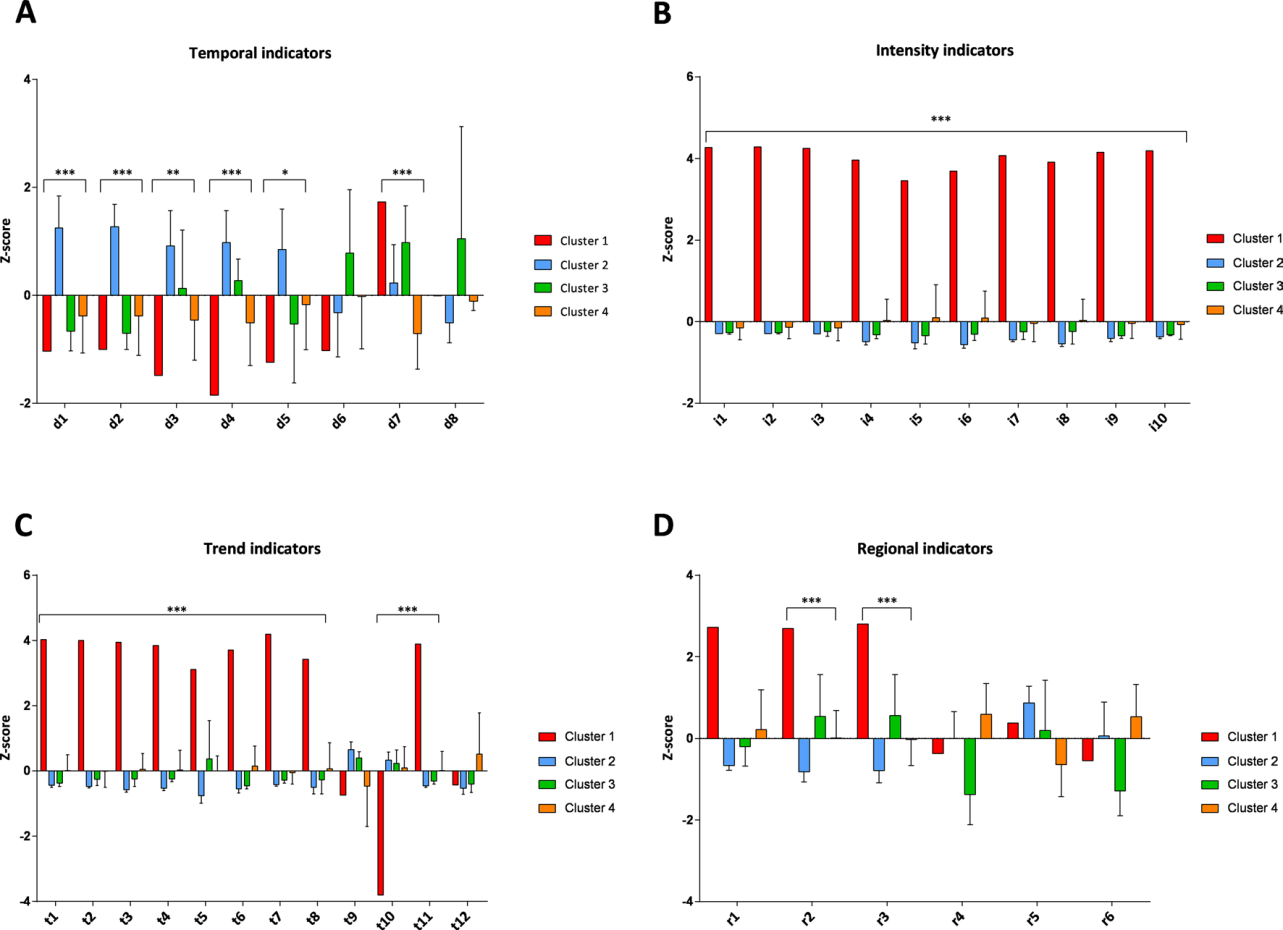
Comparison of SARS-CoV-2 epidemic indicators by clusters. This panel show the one-way analysis of variance (ANOVA) of temporal (**A**), intensity (**B**), trend (**C**), and regional (**D**) indicators across clusters. Statistical analysis was conducted after z-score standardization, and hence results can be interpreted as deviation from the national average. *p < 0.001; **p < 0.0001; ***p < 0.00001.

**Figure 3:**
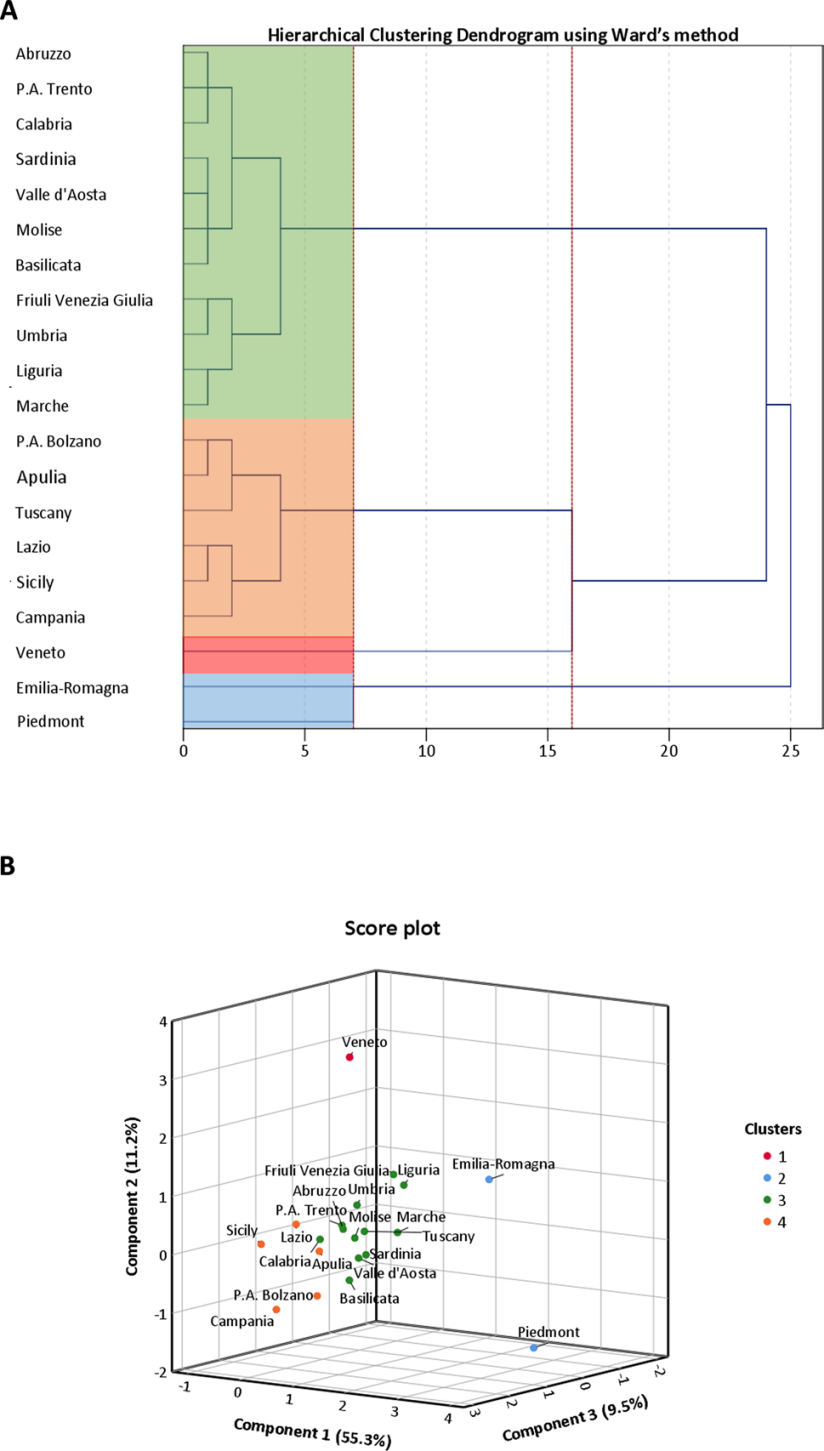
Clustering on Principal Components (PCs) after excluding Lombardy. (**A**) Dendrogram of Hierarchical Clustering based on the Ward’s criterion. The height of the branches indicates the dissimilarity between clusters. The dendrogram was partitioned (red dotted lines) to maximize the distance between nodes. Cluster solution is indicated by four colored panels. (**B**) Three-dimensional Score plot illustrating how clusters were distributed on PCs. The number of clusters to be retained was set according to the dendrogram and cluster solution was consolidated by K-means algorithm.

**Figure 4:**
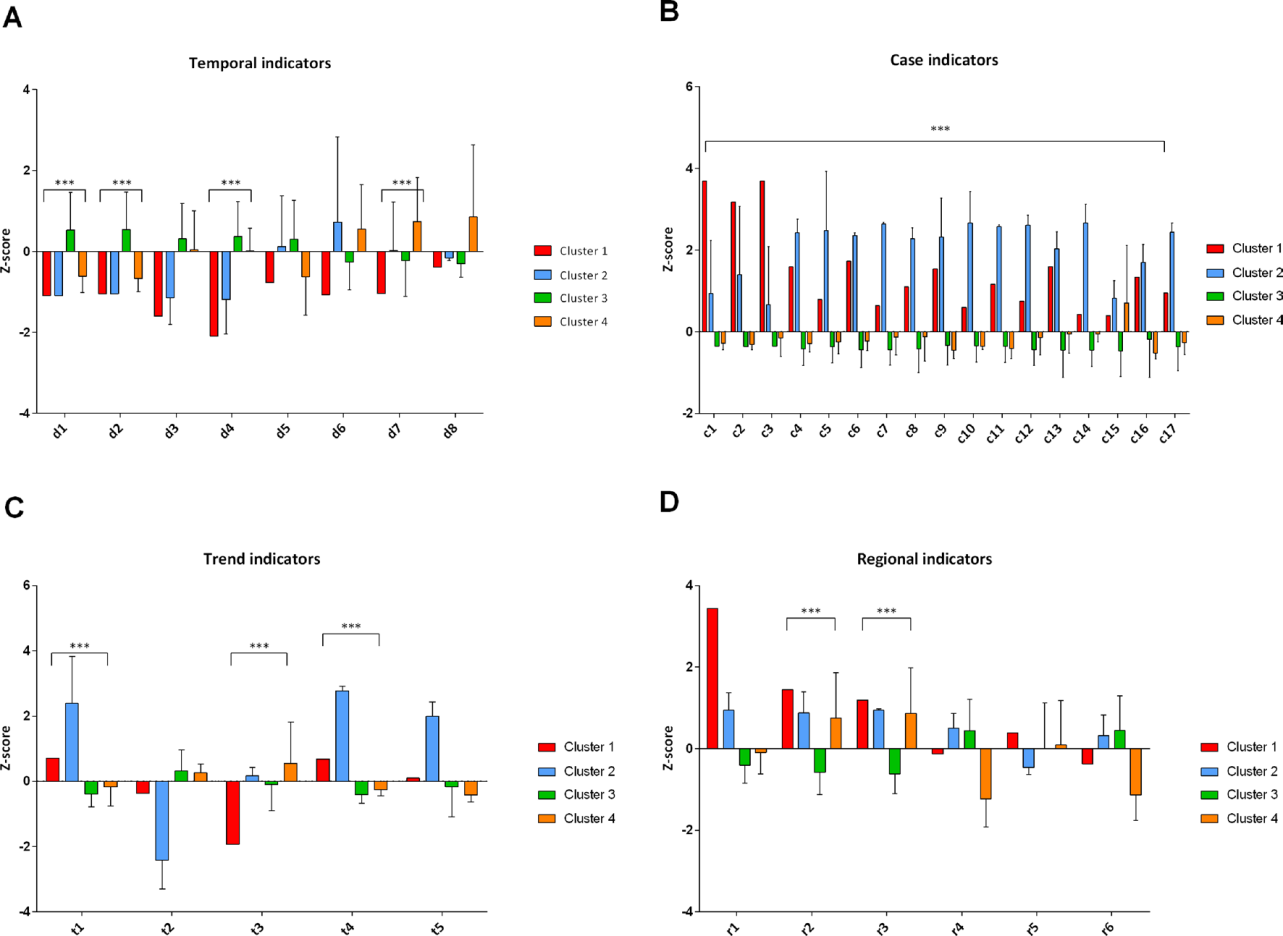
Comparison of SARS-CoV-2 epidemic indicators by clusters, after excluding Lombardy. This panel show the one-way analysis of variance (ANOVA) of temporal (**A**), intensity (**B**), trend (**C**), and regional (**D**) indicators across clusters. Statistical analysis was conducted after z-score standardization, and hence results can be interpreted as deviation from the national average (excluding data from Lombardy). *p < 0.001; **p < 0.0001; ***p < 0.00001.

Next, we compared the indicators reported in Table 1 across clusters to understand how different they are, actually. Figure 2 points out that Lombardy was the worst hit region in Italy, but also suggests that other clusters differed for some indicators related to the beginning and the course of epidemic, its intensity, and regional characteristics that might affect the epidemic itself. In line with these findings, we performed a sensitivity analysis to improve the partition of regions by excluding data from Lombardy. Using the same approach described for the entire dataset, we first reduced indicators into three uncorrelated PCs, which this time explained 76.0% of total variance (both the Scree plot and the Component plot are reported in the Supplementary Material; Figures S5, S6). In Fig. 3 we present the dendrogram and the score plot obtained from hierarchical clustering and K-means algorithm. Except for Tuscany, concordance between two clustering methods was evident. In Fig. 4 we report the comparison of clusters with respect to indicators reported in Table 1, after excluding Lombardy. To guide the readers through the comparison across regions and regional clusters, we provide trends of SARS-CoV-2 positive cases, hospitalized patients, deaths, and number of tests from 24 February to 24 April 2020 (Figures S7–S10). Accordingly, the epidemic originally started in regions belonging to clusters 1 (i.e. Veneto) and 2 (i.e. Piedmont and Emilia-Romagna), which are also those with the highest number of cases beyond Lombardy. However, while at the beginning the intensity of epidemic was higher in Veneto, it later struck Piedmont and Emilia-Romagna even more. Indeed, the latter exhibited the highest increments of SARS-CoV-2 cases, hospitalized patients and deaths, both during and at the end of the period of this study. The epidemic started to spread a while after in regions belonging to cluster 4, which included Apulia, Calabria, Campania, Lazio, Sicily and the province of Bolzano. This translated into a lower intensity of SARS-CoV-2 cases at the beginning and during the epidemic. Furthermore, regions in cluster 4 might have also benefited from younger residents and lower aging index than the other regions. In support of this, a linear regression analysis found that either age (p = 0.020) and the aging index (p = 0.039) were positively associated with the number of SARS-CoV-2 cases on 24 April, independent of the starting date of the epidemic, the number of cases on 24 February, the number of residents, the number of tests, and the proportion of men. With some exceptions (e.g. Tuscany), the remaining regions are those with lower number of residents, and therefore those with less availability of ICU beds and less tests performed. These regions are also those where the epidemic started later, and where it spread with the lowest intensity.

## Discussion

Our results provide evidence on how the SARS-CoV-2 epidemic struck Italian regions with different patterns. We applied a hierarchical clustering on PCs approach, which combined three data mining methods—namely PCA, hierarchical clustering and K-means algorithm—to provide a satisfactory clustering based on a set of epidemic indicators defined a priori*.* Although this may seem a mere statistical exercise, it has allowed to give an early snapshot of the epidemic emergency within Italian territory. Indeed, previous studies have already showed that several regional and local factors might differently affect the epidemic spread and the response to control measures across Italian regions. For instance, Lilleri and colleagues demonstrated that epidemic intensity was negatively correlated with the distance from the epidemic epicentre and positively with the regional gross domestic product. In addition, the authors showed that an earlier lockdown and a wider testing strategy were associated with a reduced number of cases^9^. Consistently, Della Rossa and colleagues stated that a better comprehension of heterogeneity between Italian regions was essential to design effective strategies against the SARS-CoV-2 epidemic. Indeed, after modelling Italy as a network of regions, the authors concluded that intermittent regional strategies might avoid saturation of regional health systems and mitigate the economic impact^10^. In a recent work, we proposed that the classification of Italian regions into different clusters could have supported the development of specific strategies after the Italian national lockdown^14^. In part this happened after 6 November 2020, when a governmental decree classified Italian regions and autonomous provinces into three areas—red, orange, and yellow—which corresponded to three different risk scenarios.

Referring back to the current study, the first advantage of our approach is that it involves the application of objective clustering techniques to the PCA outcomes, which leads to an improved cluster solution. The second advantage is the possibility of exploiting a mixed algorithm for the clustering process—a combination of the Ward’s classification method with the K-means algorithm—which improves the robustness of findings. Furthermore, our approach relies on several indicators that we have defined a priori to discriminate different clusters across Italian regions. However, it would be possible to make some variations and use it in different context or applications. Our approach has first discriminated four clusters of regions, which diverged for some indicators related to the epidemic spread, its intensity, and differences between regions that might affect the epidemic itself. Among the Italian regions however, one in particular stood out, the Lombardy, whose number of SARS-CoV-2 cases represented more than one third of the total Italian cases^2^. The first Italian case, indeed, was diagnosed in Lombardy on 20 February, 2020, when a young man was admitted with an atypical pneumonia that later proved to be caused by SARS-CoV-2^1^. In the next days, the epidemic has spread alarmingly through the region, with 171 more cases as of 24 February. On this date, just four regions—namely Veneto, Email-Romagna, Piedmont, and Lazio—reported other cases of SARS-CoV-2 infection^2^.

For this reason, we decided to exclude the Lombardy region from further analysis, in which we aimed to provide a better cluster solution. Accordingly, the remaining regions were partitioned into four clusters: cluster 1 (i.e. Veneto); cluster 2 (i.e. Piedmont and Emilia-Romagna); cluster 3 (i.e. Abruzzo, Basilicata, Calabria, Friuli Venezia Giulia, Liguria, Marche, Molise, Sardinia, Tuscany, Umbria, Valle d'Aosta, and the province of Trento) and cluster 4 (i.e. Apulia, Calabria, Campania, Lazio, Sicily and the province of Bolzano). This partition confirms that Emilia-Romagna, Piedmont, and Veneto are the most hit regions beyond Lombardy. However, while the number of SARS-CoV-2 cases was higher in Veneto on 24 February, it then increased more rapidly and with more intensity in Emilia-Romagna and Piedmont. Although the government measures were effective to slow down the epidemic in all the Italian regions^5, 6^, the comparison between cluster 1 and 2 corroborates that the earlier the measures were taken, the lower the cumulative incidence achieved^8^. Indeed, Veneto imposed regional measures of travel restrictions earlier than Emilia-Romagna and Piedmont^3^. Our results also reflect the level of attention on the epidemic and the number of tests performed over the population, which probably varied across regions. While Veneto started testing all residents who had come into contact with documented SARS-CoV-2 cases—even if they were not showing symptoms—other regions tested only residents who experienced more severe conditions. Looking at the data, Veneto conducted a wider testing campaign (approximately 44 tests per 1000 residents) if compared with Emilia-Romagna and Piedmont (24 tests per 1000 residents and 17 tests per 1000 residents, respectively)^2^. If on the one hand performing an insufficient number of tests underestimates the transmission rate and distorts the statistics^17, 18^, on the other hand combining the restrictions with widespread testing may have contributed to a more rapid resolution of the epidemic in Veneto^8^.

We also found a cluster of regions (i.e. cluster 4) where the epidemic started to spread slightly after Veneto, Emilia-Romagna and Piedmont. This has certainly contributed to the lower intensity of the epidemic among these regions, but it has also favored the efficacy of restrictive measures, which acted promptly. Our approach, however, has also uncovered one of the peculiarities of cluster 4. These regions, in fact, are among the youngest in Italy, with an aging index that ranges from 123 to 169 individuals ≥ 65 years per 100 individuals < 14 years^2^. It was demonstrated that SARS-CoV-2 infection is more severe among people aged 65 years or older^17^, so the younger age distribution in these regions might partially explain the lower epidemic intensity compared with other regions. To corroborate our hypothesis, we demonstrated that both age and the aging index were positively associated with the number of documented SARS-CoV-2 cases on 24 April, independent of other epidemic features.

The remaining regions were included in a more heterogeneous cluster (i.e. cluster 3), which—with some exception—comprised those regions where the epidemic started later and/or where it spread with the lowest intensity. Tuscany, actually, departed slightly from the other regions in cluster 3, assuming some characteristics typical of cluster 4. The uncertainty in assigning Tuscany into one of two clusters is probably due to hybrid characteristics of this region, which indeed was the only to have received different allocations depending on the clustering method.

Our study has some limitations to be considered. First, it does not take into the proportion of undocumented events, which might differently affect some indicators^18, 19^. Our approach, furthermore, considers only a part of the availability of medical care resources, indicated in terms of ICU beds. Further analyses should include additional indicators of the healthcare system that might have influenced the response to the epidemic^20^. Indeed, our clustering must not be seen as a fixed approach, but it could be integrated and modified, as experience and knowledge on the SARS-CoV-2 epidemic increase. For instance, the cumulated and weighted average daily growth rate proposed by Bartolomeo and colleagues could represent an additional and/or alternative indicator to be included in the clustering algorithm^21^. With these considerations in mind, we cannot completely exclude the potential effect of unmeasured factors and further research should be encouraged to refine our model.

In conclusion, our findings provide policymakers with a snapshot of the current epidemic in Italy, region by region. Distinguishing different clusters of epidemic patterns is important to assess the efficacy of restrictions imposed by the Italian Government. This delineation might also help guiding the upcoming countermeasures, which should be adopted in accordance with the situation at regional level. Furthermore, appropriate changes to our approach could make it useful to manage this emergency also in those countries where the epidemic is still in the early stages.

## Methods

We first defined a set of 36 indicators of SARS-CoV-2 epidemic in Italy (Table 1), which reflected different domains, including: (i) the distribution of infections and related events along the temporal axis (i.e. temporal indicators), (ii) the epidemic intensity across Italian regions (i.e. intensity indicators), (iii) trend of events (i.e. trend indicators), and (iv) regional characteristics that might affect the epidemic and data reporting (i.e. regional indicators). Specifically, we used the following sources of data to extract indicators for each of the Italian regions:

- daily data on documented SARS-CoV-2 cases (including the number of infections, hospitalizations, deaths and recovered patients) released by the Italy’s Civil Protection of the Italian Ministry of Health from 24 February to 24 April, 2020^2^.
- data on the availability of Intensive Care Unit (ICU) beds across Italian Regions, released by Italian Ministry of Health in 2019 and referred to 2017^22^;
- data on the number of residents, mean age, proportion of men, and aging index reported by the Italian National Institute of Statistics (ISTAT, Istituto Nazionale di Statistica-Italian National Institute of Statistics) and referred to 1 January, 2019^23^. Among these, the aging index referred to the number of individuals aged 65 years and over per 100 individuals younger than 14 years old^23^.

After checking the normality of each indicator through the Kolmogorov–Smirnov test, all indicators were standardized using the Z-Score formula to account for different scales. The degree of correlation between indicators was examined with correlation matrix based on Pearson's correlation analysis. We next employed a PCA to reveal the underlying structure of the data. PCA is an unsupervised learning method that simplifies the complexity in high-dimensional dataset while retaining trends and patterns. This was important for working with our dataset, where a lot of variables were correlated with each others. For instance, the number of residents and the number of tests for SARS-CoV-2 might widely affect the other indicators. Specifically, PCA works by reducing the dataset into fewer dimensions called Principal Components (PCs), which are uncorrelated with each other^15^. For each PC, the eigenvalue represents the total amount of variance explained, while the eigenvector represents its orientation. The number of PCs to be retained is usually set according to eigenvalues examination through the Scree plot and variance explained^24, 25^. Accordingly, we retained those PCs with eigenvalues ≥ 3. PCs can be interpreted in terms of correlations with initial variables, which are represented by the component loadings depicted in the Component plot. To simplify the interpretability of PCs, the varimax rotation (i.e. an orthogonal rotation that minimizes the number of variables that have high loadings on each PC) was applied^26–28^. Finally, individual scores were generated for each PC and plotted in a Score plot.

We next applied a hierarchical clustering on PCs to choose the number of clusters based on the hierarchical tree. In this clustering, the nodes start off as objects and are then iteratively merged based on pairwise distance. Although there are many ways of calculating this distance, we used the Ward’s criterion because it is based on the multidimensional variance like PCA. Clustering is usually shown by a dendrogram, where the height of the branches indicates the distance or dissimilarity between clusters^11^. Here, we determined the number of clusters by partitioning the dendrogram to maximize the distance between nodes^11^.

In contrast to hierarchical clustering, k-means clustering requires a predefined number of clusters. In brief, the algorithm partitions *n* observations into *k* clusters by minimizing within-cluster variances, expressed as squared Euclidean distances to the nearest “centroids”^11^. Here, we consolidated the initial partition by using the k-means algorithm with the number of clusters defined through the hierarchical clustering. It is worth mentioning that slight differences in the clustering outcome could be obtained using the two methods^11^. Finally, we checked cluster quality, in terms of within-cluster and between-cluster variability, using the one-way analysis of variance (ANOVA). We also conducted a sensitivity analysis by excluding data from Lombardy, the region with the highest number of SARS-CoV-2 cases in Italy.

All the analyses were performed on the SPSS software (version 23.0, SPSS, Chicago, IL, USA), with a Bonferroni-corrected significance level α of 0.001.

## Supplementary Information

Supplementary Information.

## Data Availability

Data used in this study are publicly available.

## References

[1] Day M (2020). Covid-19: Italy confirms 11 deaths as cases spread from north. BMJ.

[2] Italian Ministry of Health. Covid-19. Situation report update at 24 April 18:00, http://www.salute.gov.it/portale/nuovocoronavirus/homeNuovoCoronavirus.jsp?lingua=english (2020).

[3] Italian Ministry of Health. Novel coronavirus, http://www.salute.gov.it/portale/nuovocoronavirus/homeNuovoCoronavirus.jsp?lingua=english (2020).

[4] Signorelli C, Scognamiglio T, Odone A (2020). COVID-19 in Italy: Impact of containment measures and prevalence estimates of infection in the general population. Acta Biomed..

[5] Giordano G (2020). Modelling the COVID-19 epidemic and implementation of population-wide interventions in Italy. Nat. Med..

[6] Maugeri A, Barchitta M, Battiato S, Agodi A (2020). Modeling the novel coronavirus (SARS-CoV-2) outbreak in Sicily, Italy. Int. J. Environ. Res. Public Health.

[7] Maugeri A, Barchitta M, Battiato S, Agodi A (2020). Estimation of unreported SARS-CoV-2 cases in Italy using a Susceptible-Exposed-Infectious-Recovered-Dead model. J. Glob. Health.

[8] Sebastiani G, Massa M, Riboli E (2020). Covid-19 epidemic in Italy: Evolution, projections and impact of government measures. Eur. J. Epidemiol..

[9] Lilleri D, Zavaglio F, Gabanti E, Gerna G, Arbustini E (2020). Analysis of the SARS-CoV-2 epidemic in Italy: The role of local and interventional factors in the control of the epidemic. PLoS ONE.

[10] Della Rossa F (2020). A network model of Italy shows that intermittent regional strategies can alleviate the COVID-19 epidemic. Nat. Commun..

[11] Altman N, Krzywinski M (2017). Clustering. Nat. Methods.

[12] Di Castelnuovo A (2020). Common cardiovascular risk factors and in-hospital mortality in 3,894 patients with COVID-19: Survival analysis and machine learning-based findings from the multicentre Italian CORIST Study. Nutr. Metab. Cardiovasc. Dis..

[13] Noor FM, Islam MM (2020). Prevalence and associated risk factors of mortality among COVID-19 patients: A meta-analysis. J. Community Health.

[14] Maugeri A, Barchitta M, Agodi A (2020). A clustering approach to classify Italian regions and provinces based on prevalence and trend of SARS-CoV-2 cases. Int. J. Environ. Res. Public Health.

[15] Lever J, Krzywinski M, Altman N (2017). Principal component analysis. Nat. Methods.

[16] Husson, F., Josse, J. & Pagès, J. *Principal Component Methods—Hierarchical Clustering—Partitional Clustering: Why Would We Need to Choose for Visualizing Data?* (2010).

[17] Onder G, Rezza G, Brusaferro S (2020). Case-fatality rate and characteristics of patients dying in relation to COVID-19 in Italy. JAMA.

[18] Maugeri A, Barchitta M, Battiato S, Agodi A (2020). Estimation of unreported novel coronavirus (SARS-CoV-2) infections from reported deaths: A susceptible-exposed-infectious-recovered-dead model. J. Clin. Med..

[19] Tuite AR, Ng V, Rees E, Fisman D (2020). Estimation of COVID-19 outbreak size in Italy. Lancet Infect. Dis..

[20] Ji Y, Ma Z, Peppelenbosch MP, Pan Q (2020). Potential association between COVID-19 mortality and health-care resource availability. Lancet Glob. Health.

[21] Bartolomeo N, Trerotoli P, Serio G (2021). Short-term forecast in the early stage of the COVID-19 outbreak in Italy. Application of a weighted and cumulative average daily growth rate to an exponential decay model. Infect. Dis. Model.

[22] Italian Ministry of Health. Annuario Statistico del Servizio Sanitario Nazionale, http://www.salute.gov.it/imgs/C_17_pubblicazioni_2879_allegato.pdf (2019).

[23] Istituto Nazionale di Statistica, ISTAT. https://www.istat.it/en/.

[24] Agodi A (2018). Association of dietary patterns with metabolic syndrome: Results from the Kardiovize Brno 2030 Study. Nutrients.

[25] Barchitta M (2019). Dietary patterns are associated with leukocyte LINE-1 methylation in women: A cross-sectional study in southern Italy. Nutrients.

[26] Barchitta M (2018). The association of dietary patterns with high-risk human papillomavirus infection and cervical cancer: A cross-sectional study in Italy. Nutrients.

[27] Maugeri A (2019). How dietary patterns affect left ventricular structure, function and remodelling: Evidence from the Kardiovize Brno 2030 study. Sci. Rep..

[28] Maugeri A (2019). Maternal dietary patterns are associated with pre-pregnancy body mass index and gestational weight gain: Results from the "Mamma & Bambino" cohort. Nutrients.

