# Supplementary material for: Applying a hierarchical clustering on principal components approach to identify different patterns of the SARS-CoV-2 epidemic across Italian regions
Source: Sci Rep. 2021 Mar 29;11:7082. doi: 10.1038/s41598-021-86703-3 (PMC8007710; doi:10.1038/s41598-021-86703-3)

SUPPLEMENTARY FILES

Applying a Hierarchical Clustering on Principal Components Approach to Identify Different Patterns of the SARS-CoV-2 Epidemic across Italian Regions

Andrea Maugeri [1], Martina Barchitta [1], Guido Basile [2] and Antonella Agodi [1,3,*]

[1] Department of Medical and Surgical Sciences and Advanced Technologies "GF Ingrassia", University of Catania, 95123, Catania, Italy

[2] Department of General Surgery and Medical-Surgical Specialties, University of Catania, 95123 Catania, Italy

[3] Azienda Ospedaliero-Universitaria "Policlinico-Vittorio Emanuele", 95123 Catania, Italy

* Correspondence to Antonella Agodi; Department of Medical and Surgical Sciences and Advanced Technologies "GF Ingrassia", University of Catania, 95123, Catania, Italy; email address: agodia@unict.it; telephone number: +39 095 3782183

Figure S1. Correlation matrix of indicators defined *a priori* to characterize the SARS-CoV-2 epidemic across Italian regions. This matrix intends to summarize correlation coefficients between indicators described in **Table 1**. Indicators are strictly correlated with each other, with some exceptions (e.g. d6, d8, t12, r4, r5, and r6). Results are obtained after z-score standardization and reported as Pearson correlation coefficients.

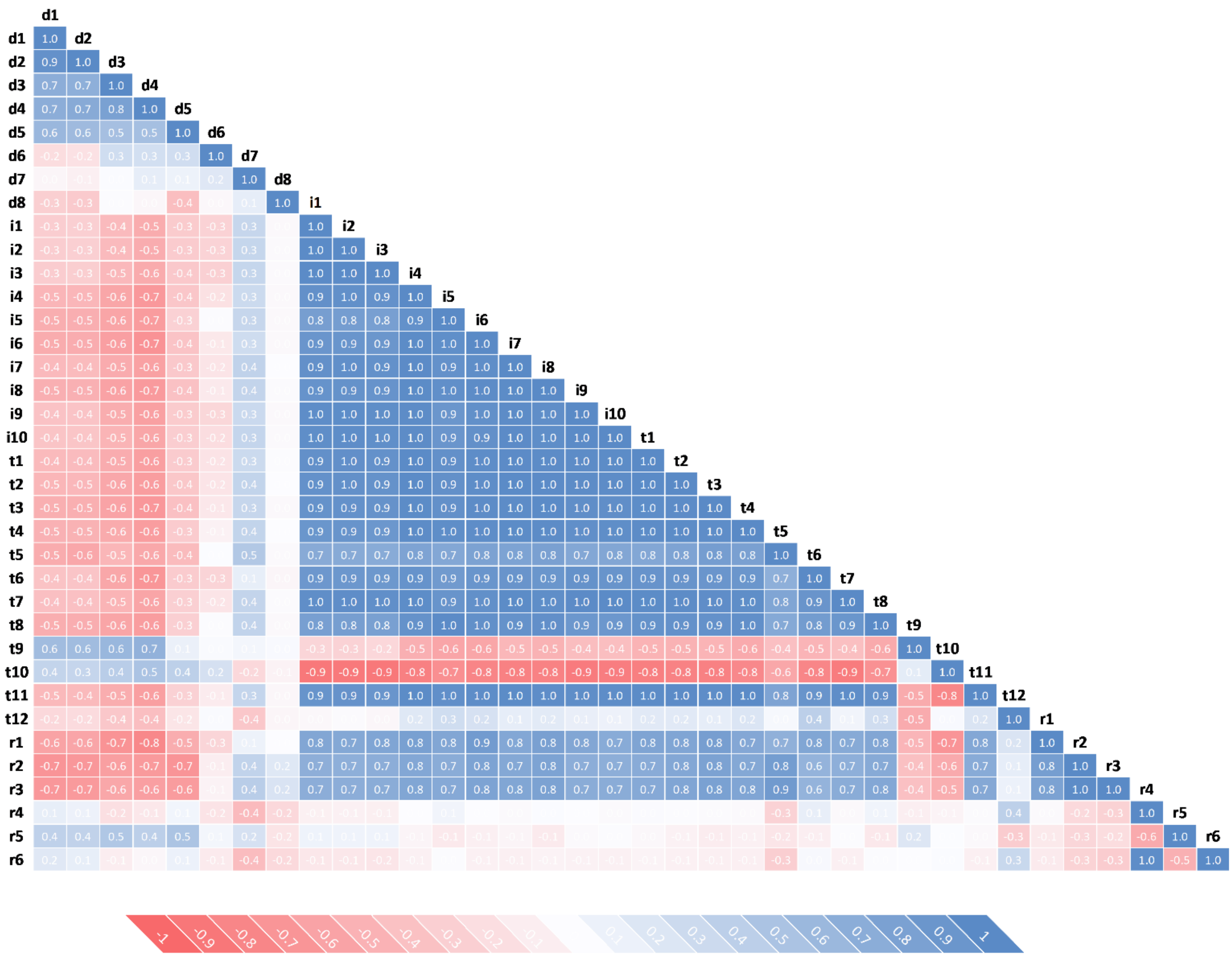

Figure S2. Scree plot of Principal Component Analysis

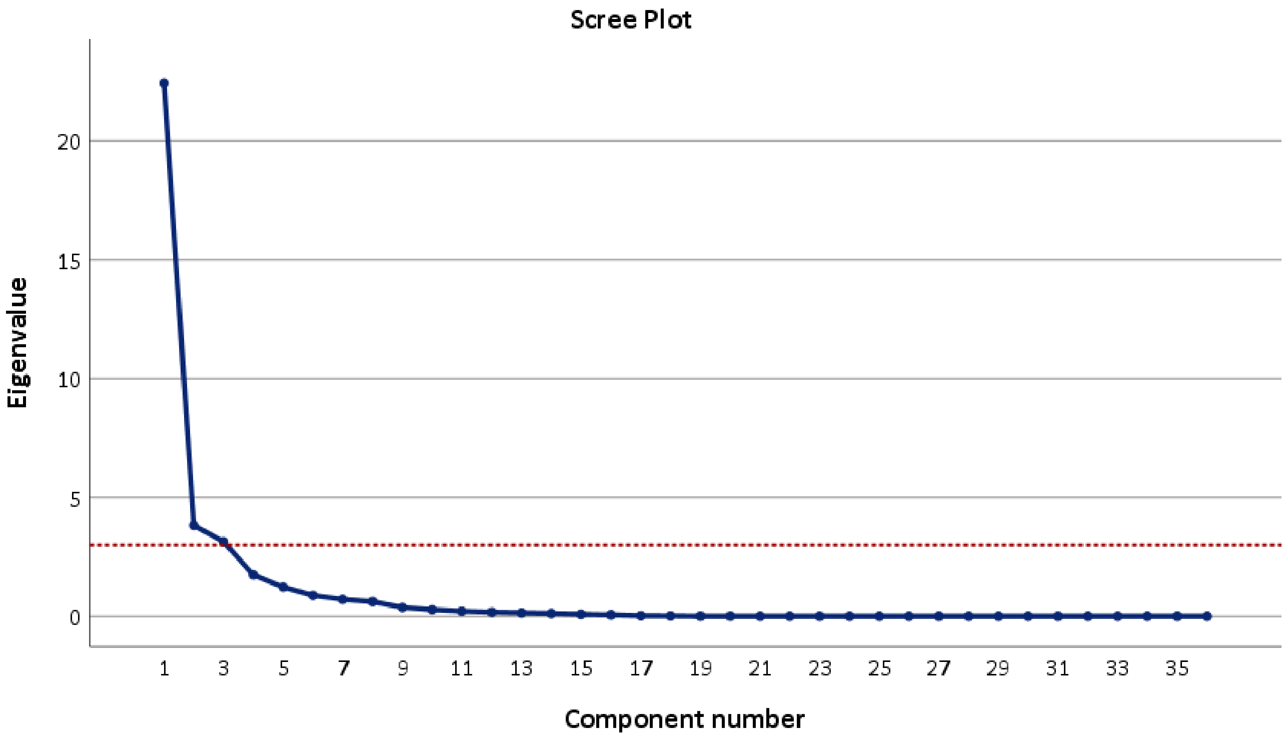

Figure S3. Outputs of Principal Component Analysis (PCA). (A) Component plot and (B) Score plot of PCA of indicators defined a priori to characterize the SARS-CoV-2 epidemic across Italian regions. PCA followed by varimax rotation was employed to reveal the underlying structure of the data and to reduce the dataset into principal components (PCs), which were uncorrelated with each other. The number of PCs to be retained was chosen according to eigenvalues ≥ 3 and Scree plot examination (Fig. S1). (A) Plot of component loadings which can be interpreted as correlations with initial variables. PC1 explained 62·3% of total variance and correlated with intensity, trend and regional indicators; PC2 explained 10·6% of variance and was negatively correlated with temporal indicators and positively with regional indicators; PC3 explained 8·7% of variance and correlated with regional indicators. (B) Three-dimensional Score plot illustrating how Italian regions were distributed on PCs.

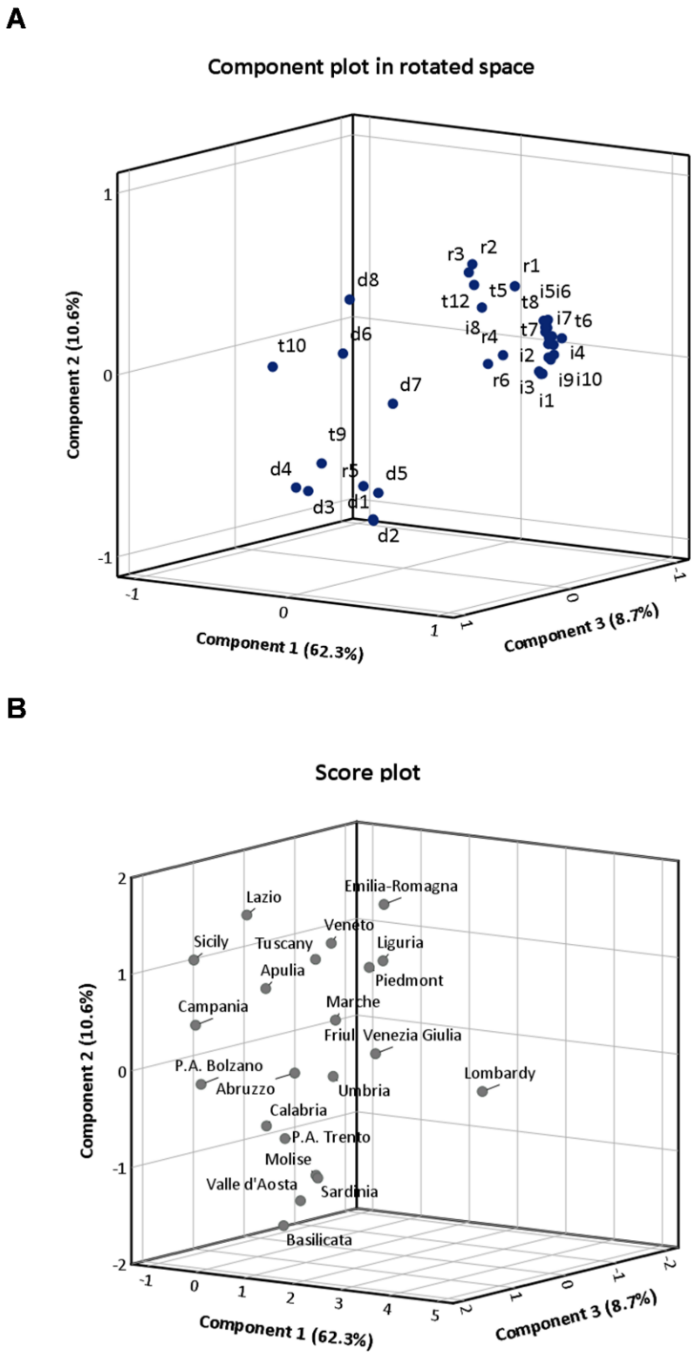

Figure S4. Three-dimensional Score plot illustrating how Italian regions were distributed on PCs obtained from the dataset with indicators normalized to the number of residents.

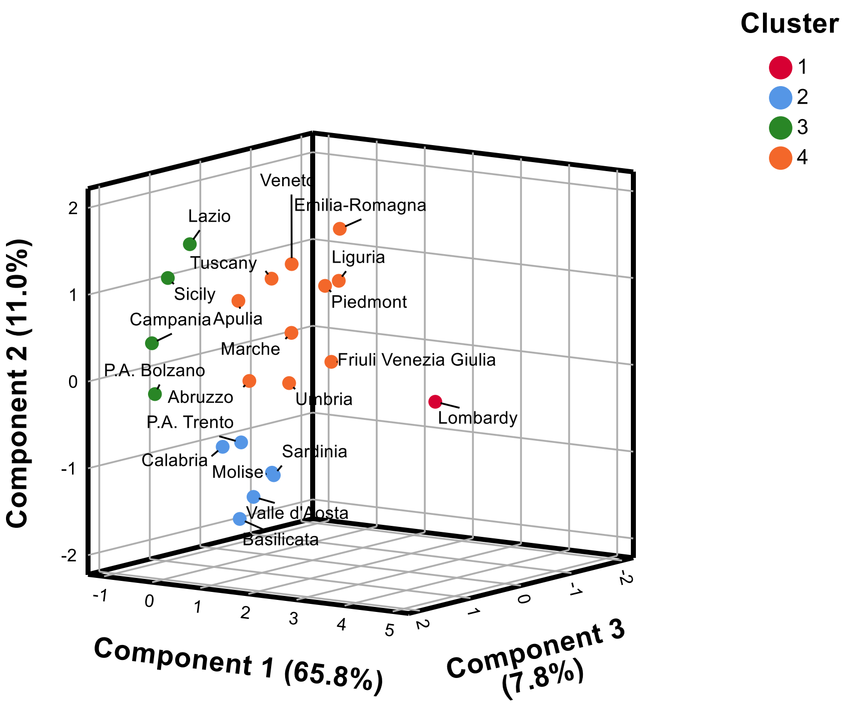

Figure S5. Scree plot of Principal Component Analysis after excluding Lombardy

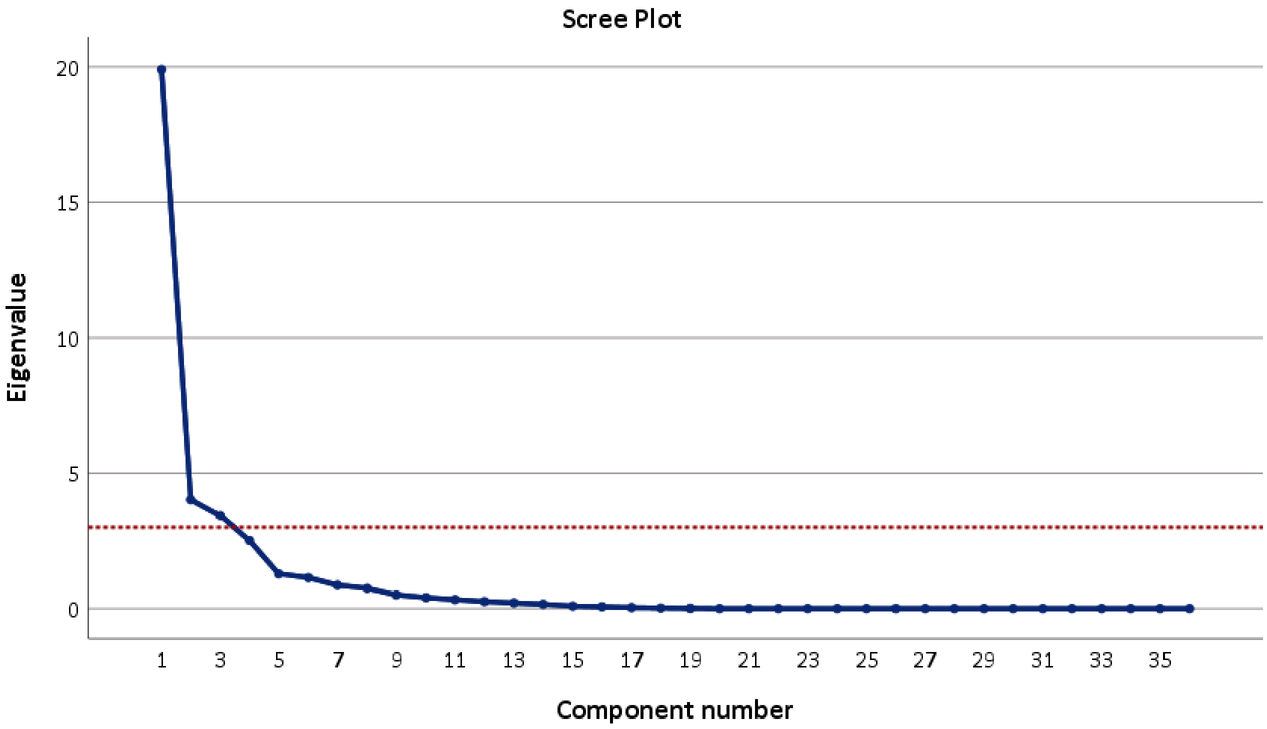

Figure S6. Component plot of Principal Component Analysis after excluding Lombardy

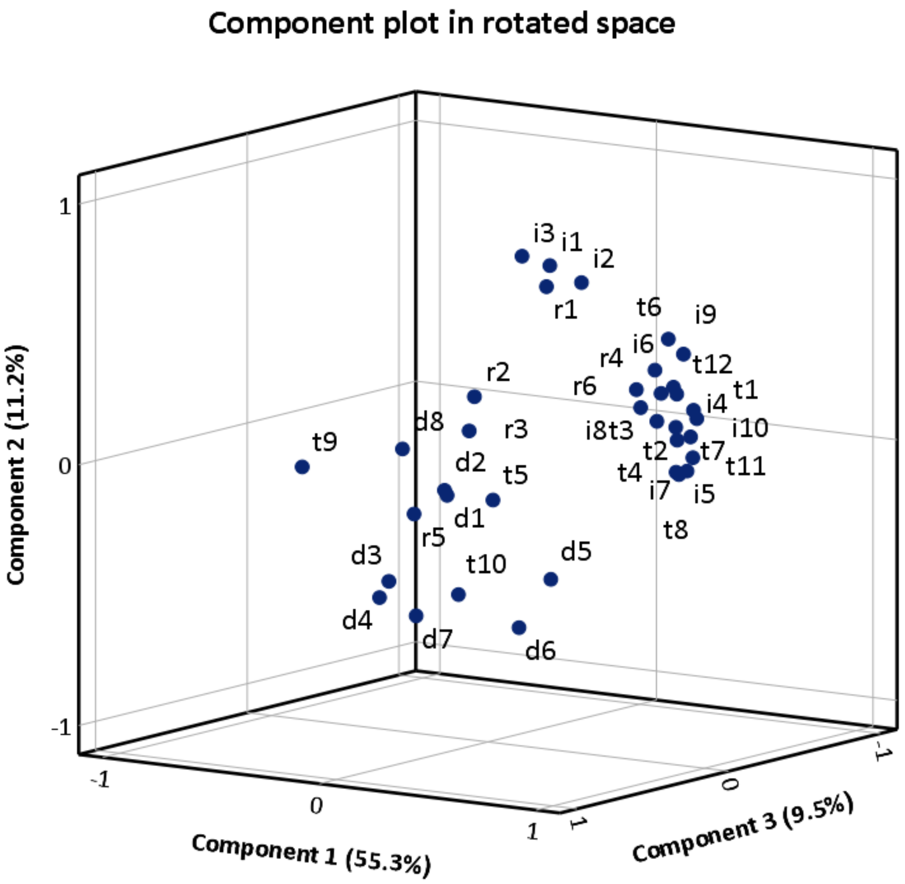

Figure S7. Regional trends of SARS-CoV-2 positive cases from 24 February to 24 April 2020

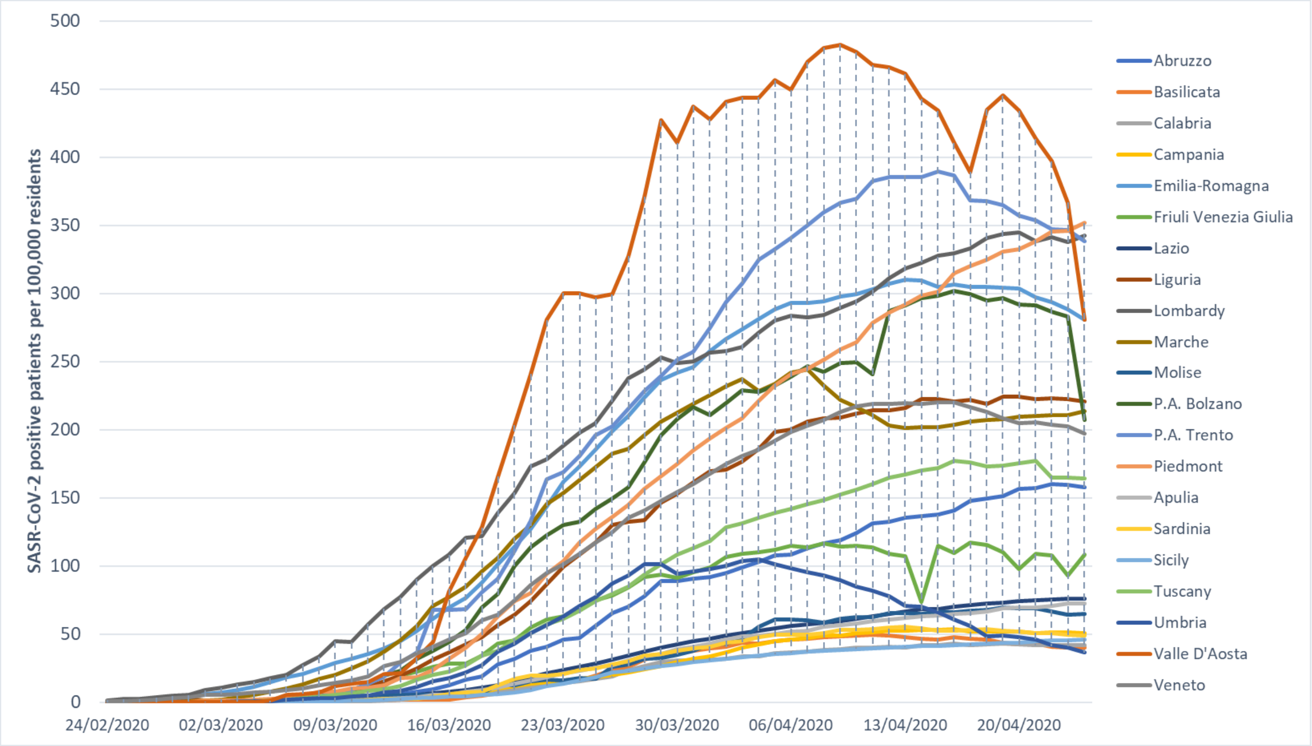

Figure S8. Regional trends of hospitalized patients from 24 February to 24 April 2020

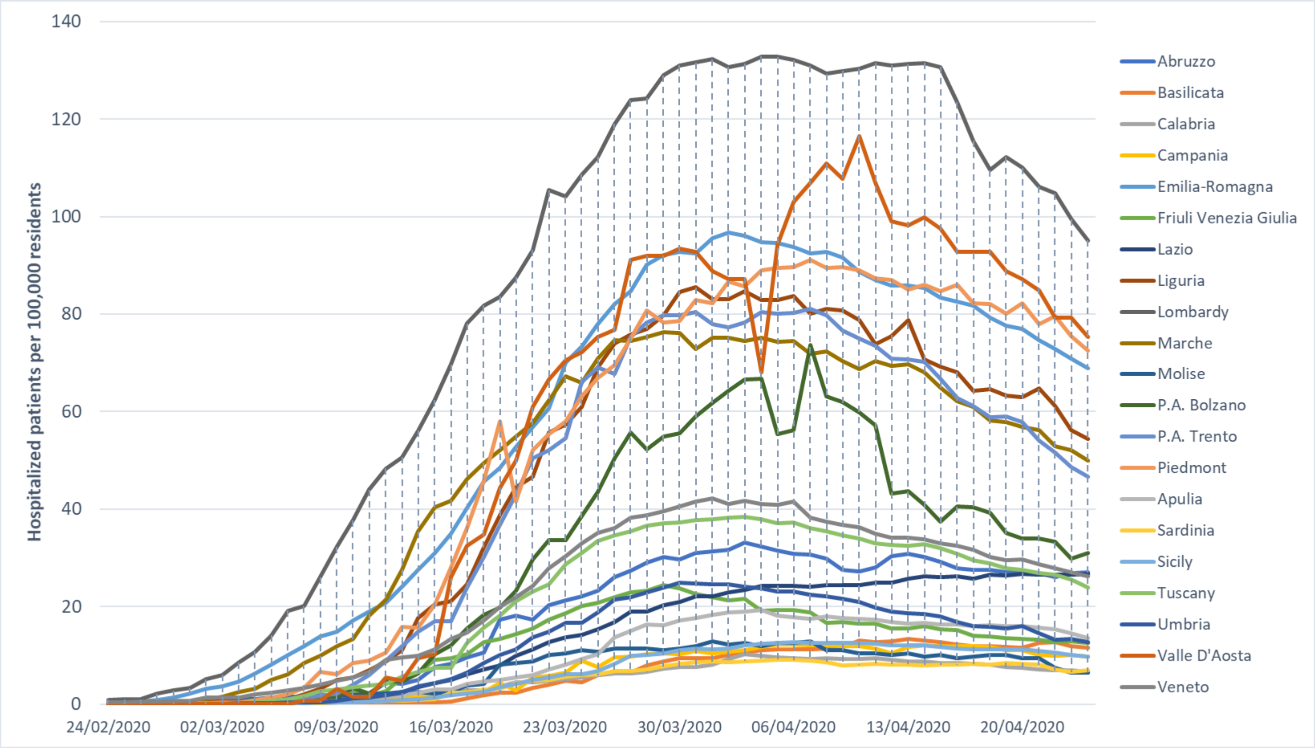

Figure S9. Regional trends of deaths from 24 February to 24 April 2020

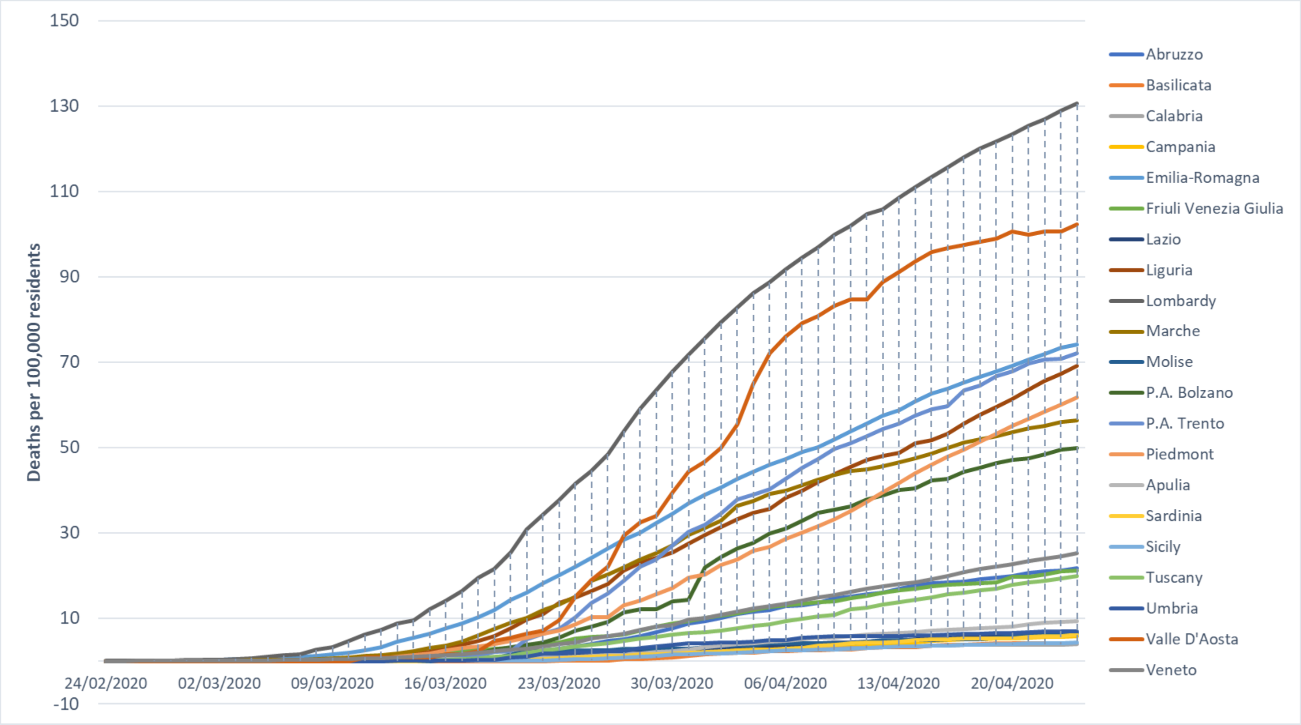

Figure S10. Regional trends of test for SARS-CoV-2 from 24 February to 24 April 2020

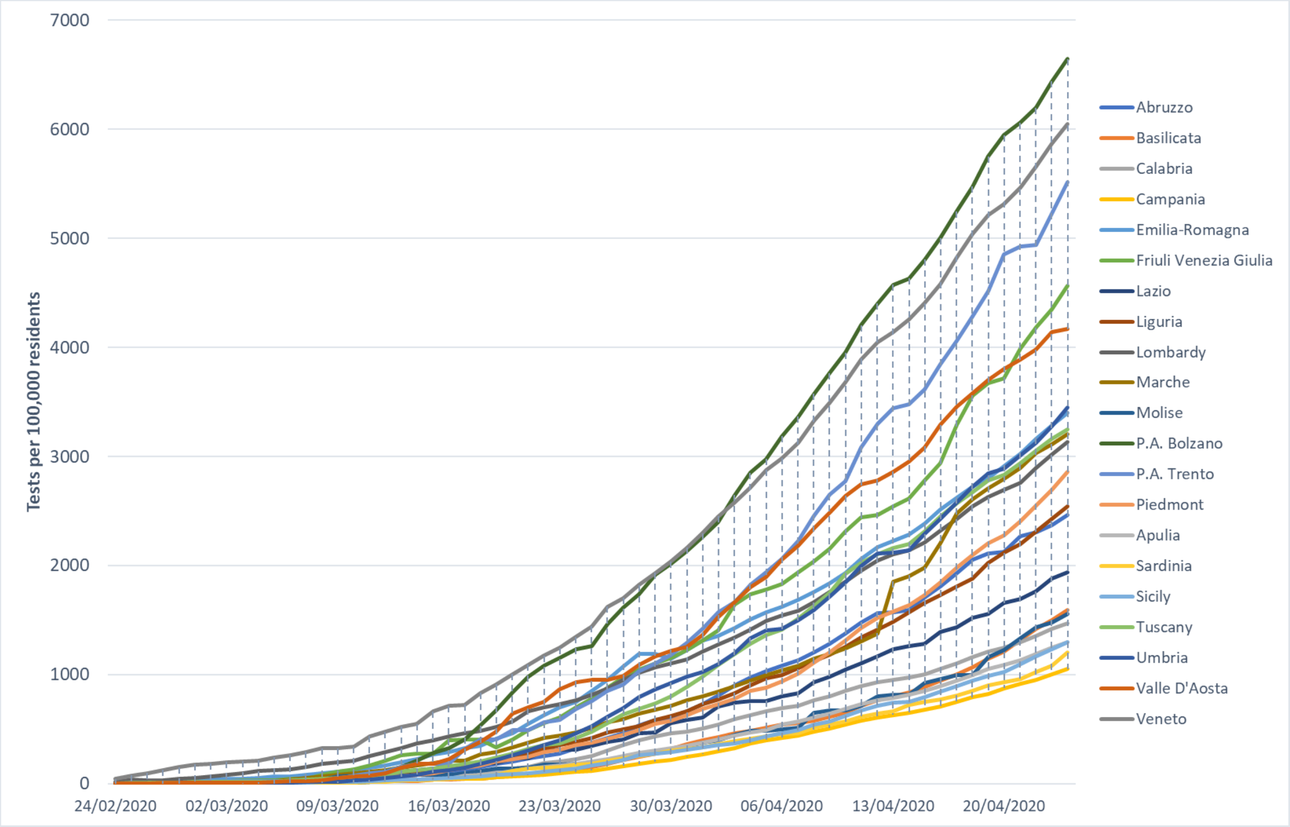

Supplement: Supplementary file 1 — Supplementary Information. [file 41598_2021_86703_MOESM1_ESM.pdf]
